# Supplementary material for: Complexome profiling on the Chlamydomonas lpa2 mutant reveals insights into PSII biogenesis and new PSII associated proteins
Source: J Exp Bot. 2021 Aug 26;73(1):245–62. doi: 10.1093/jxb/erab390 (PMC8730698; doi:10.1093/jxb/erab390)
Supplement: erab390_suppl_Supplementary_Dataset_S1 [file erab390_suppl_supplementary_dataset_s1.zip › Supplemental Dataset 1 - Excel List and all profiles/plots/NUO8_Cre12.g496750.html]

Trivial name: NUO8  
  
Euclidean distance: 108721.49  
Mean Intensity (WT): 10938.72  
Mean Intensity (Mut): 9065.19  
Distance: 9.94  
  
MapMan: mitochondrial electron transport / ATP synthesis.NADH-DH (type I).localisation not clear;mitochondrial electron transport / ATP synthesis.NADH-DH (type I).complex I  
  
p value of intensity sums Welch test: 0.5925
